# Composition and antioxidant activity of *Abies marocana* essential oil

**DOI:** 10.1038/s41598-026-38235-x

**Published:** 2026-02-02

**Authors:** Mohamed El Bakkali, Amin Bouchfara, Hamass Zerrad, Badredine Souhail, Mounir Nechar, Rehana Bano, Béla Fiser

**Affiliations:** 1 Laboratory of Water, Studies and Environmental Analysis, Faculty of Sciences, Abdelmalek Essaâdi University, Tétouan, Morocco; 2 Biotechnologies and Biomolecular Engineering Research Team, Faculty of Sciences and Technologies, Abdelmalek Essaâdi University, Tangier, Morocco; 3 Higher Education and Industrial Cooperation Centre, University of Miskolc, Miskolc-Egyetemváros, 3515 Hungary; 4 Institute of Chemistry, University of Miskolc, Miskolc-Egyetemváros, 3515 Hungary; 5 Department of Physical Chemistry, Faculty of Chemistry, University of Lodz, 90-236 Lodz, Poland

**Keywords:** Abies marocana, AMEO, Phytochemical, Physicochemical properties, Molecular docking, Antioxidant activity, Biochemistry, Chemical biology, Chemistry, Drug discovery, Plant sciences

## Abstract

Belonging to the Pinaceae family *Abies marocana* is a native tree from calcareous mountains of Jebala in northern Morocco. This endemic species is distributed over Mont Tazaout and Talassemtane in the province of Chefchaouen. The present study explores for the first time the phytochemical composition, physicochemical properties, total phenolic content (TPC) and molecular docking simulations to evaluate antioxidant activity of Mont Tazaout *Abies marocana* essential oil (AMEO). AMEO was extracted using steam distillation, with a yield of 0.24%. A total of 49 compounds were identified using gas-chromatography coupled with mass spectrometry (GC/MS) method. Monoterpene hydrocarbons (88.55%) being the dominant class, and the main components of the tested oil were limonene (38.46%), *α-*Pinene (21.16%), *β*-Pinene (12.92%) and camphene (11.23%). Physicochemical properties of AMEO were determined, including density (0.888 ± 0.020) g/cm^3^, refractive index (1.470 ± 0.010), and optical rotation (-40.52 ± 0.04)˚. Furthermore, the total phenolic content (TPC) of the essential oil was found to be (5.07 ± 0.49) mg GAE/g EO. AMEO shows an IC_50_ = (136.28 ± 7.8) mg/mL and IC_50_ = (1.47 ± 0.04) mg/mL in the DPPH and FRAP assays, respectively. Molecular docking used to investigate the mechanism of interaction between the constituents of AMEO and target proteins associated with antioxidant activity. After analyzing the docking results related to the docking of the seven studied compounds (*S*-limonene, *R*-limonene, camphene, (+)-*α*-pinene, (-)-*α*-pinene, (+)-*β*-pinene and (-)-*β*-pinene), compounds *R*-limonene and *S*-limonene emerged as the most promising antioxidants. This is due to their favorable binding profiles with both NAD(P)H oxidase and nitric oxide synthase targets. These findings highlight the potential bioactive properties of AMEO, suggesting its relevance for further exploration in pharmaceutical, cosmetic and medicinal food reformulation.

## Introduction

 Plants have received considerable attention for their role in traditional medicine, with many reported to possess diverse biological activities that could offer therapeutic benefits. Essential oils are volatile secondary metabolites of medicinal plants, are present in various parts of these plants, including seeds, leaves, flowers, fruits, stems, and roots. Essential oils are well-known for their diverse biological activities, particularly their antimicrobial, antifungal, and antioxidant properties^1^. Environmental factors play an important role in the production and accumulation of secondary metabolites in medicinal plants. Key factors such as temperature, rainfall, light intensity, and altitude, which define the climate of a region, significantly influence the accumulation of these metabolites. Research has demonstrated the efficacy of essential oils in reducing the risk of diseases such as diabetes, cancer, and cardiovascular disease^2^. In addition, essential oils have also demonstrated antiviral, antifungal, antitoxigenic, antiparasitic, and insecticidal effects^3,4^.

Given this broad spectrum of biological activities, particular attention has been directed toward their antioxidant potential, especially as alternatives to synthetic antioxidants widely used in the food and pharmaceutical industries^5^. In the past few years, consumers are becoming more worried about the use of manufactured additives in the food industry around the world. Synthetic antioxidants, such as butylated hydroxy anisole (BHA), butylated hydroxy toluene (BHT) and tert-butyl hydroquinone (TBHQ), are widely used in the food industry because they are effective and less expensive than natural antioxidants^6^. In contrast, natural antioxidants such as flavonoids, tannins, coumarins, curcumanoids, xanthons, phenolics, and terpenoids are found in various plant products (such as fruits, leaves, seeds, and oils). Compared to individual antioxidant substances, plant extracts containing a mixture of polyphenolic compounds offer a promising solution due to their synergistic effects^6,7^. The limitations on the use of synthetic antioxidants and the growing interest in natural, non-toxic alternatives have led to numerous studies exploring the antioxidant potential of essential oils^8^. Increasing the use of plant-derived natural antioxidants in the form of extracts requires fulfilling certain obvious requirements^9^.

The genus *Abies* is the second-largest genus is considered to be the most complex genera belongs to the Pinaceae family which has nine genera^10–12^. The genus *Abies* comprises 51 species of evergreen conifers found across the Northern Hemisphere’s temperate and boreal zones, especially in mountainous areas such as North Africa, the Himalayas and Türkiye^10,13^, occurring in mountainous throughout most of their range^14,15^. They frequently from interspecific hybrids, exhibiting significant variability in morphological, anatomical, and biochemical characteristics^16^. As reported, for the coastal regions of the Taurus Mountains certain species of *Abies* genus are attributed with medicinal properties used to treat indigestion, stomachaches, colds, as well as vascular, pulmonary, and venereal diseases^17^. Approximately 277 compounds have been identified from 19 species of *Abies* genus, primarily consisting of terpenoids, flavonoids, and lignans, along with minor constituents such as phenols, steroids, fatty acids, and fatty alcohols^18,19^. In the most cases the essential oils of *Abies* species contain α-pinene, *β*-pinene, camphene, limonene, carene, bornyl acetate, and caryophyllene as their main components^20^. The essential oils composition of *Abies* species demonstrates both shared traits and regional distinctions and environmental influences. For instance, Abies alba seeds from Poland are characterized by high concentration of limonene (82.9%) and followed by camphene (6.3%)^21^. In contrast, *Abies balsamea* branches from Canada exhibit a more diverse chemical profile by *δ*-3-carene (14%), *α*-pinene (11.3%), bornyl acetate (10%), *β*-phellandrene (7.2%), and limonene (7.1%)^22^. Meanwhile, *Abies koreana* seeds, also from Poland, display a composition that bridges characteristics of both previous samples, with limonene (53.7%) as the dominant constituent, and nearly equal levels of *α*-pinene (12.1%), bornyl acetate (12.1%), and camphene (11.2%) ^21^. These findings suggest that while limonene is a common and often abundant component across various *Abies* species, the relative presence of other compounds such as pinene isomers and bornyl acetate varies significantly by species and plant part, reflecting both genetic and environmental diversity^23^. The rosin of *Abies cilicica* from Turkey, which primarily contains car-3-ene, caryophyllene oxide, and caryophyllene, has traditionally been used for its antiseptic, anti-inflammatory, antipyretic, antibacterial, and antiviral properties. *Abies cilicica* was also been utilized as chewing gum to treat stomach ailments such as ulcers, lip dryness, and asthma, as well as for wound healing in the form of ointments and plasters^24^. The essential oils extracted from the needles of *Abies* species are commonly used in the soap and perfume industries to enhance products with their pleasant fragrances^15^. A recent discovery has led to the extraction of a compound from the bark of the silver fir tree (*Abies alba*), which has previously demonstrated antioxidant properties in studies and has now been launched in the market under the brand names Abigenol^®^ and AlbiPhenol^®^^25,26^.

Morocco, due to its climate and geographical location, is rich in coniferous trees, which account for about two-thirds of the country’s plant species^27^, *Abies marocana* (Moroccan fir) is an endemic Moroccan tree, native of the Rif region, thriving at elevations between 1,500 and 2,100 m (but appears since 1,300 as scattered trees) and able to reach 50 m in height, under cool and cold variants of humid to perhumid bioclimates^28,29^. The climate of the Rif fir forest, where *Abies marocana* naturally grows is distinctive—characterized by Atlantic influence during winter and spring, and Mediterranean influence in the summer^16^. Currently, the Rif fir forests stretch a little more than 4,000 ha distributed among two distinct locations, namely the Talassemtane forest and the Tazaout forest, which occupy an area of about 3,760 ha and approximately 300 ha respectively^16^. *Abies marocana* plays a vital role in forming stunning forest ecosystems, particularly around the peaks near the Moroccan city of Chefchaouen^13^. This species is endemic to the high-altitude calcareous peaks of the western Rif Mountains in Morocco. *Abies marocana* (needles, twigs, and cones) have been observed in pharmaceuticals due to the presence of active compounds. Needles of *Abie*s *marocana* are rich with carbohydrates, crude protein, crude fiber, crude fat, ash, and moisture^30^. Needles are a source of mineral elements and contain significant levels of phenols, flavonoids, tannins, and phytosterols. Previous phytochemical investigations of *Abies marocana* have focused on solvent extracts from the aerial parts of the plant. These aerial parts contain phenolic compounds, terpenes, essential oils, and sterols across various organs^13^. Notably, the needles and cones are richer in flavonoids compared to the twigs^13^.

Molecular docking was successfully employed to predict the positioning (orientation and conformation) of a ligand in a target interaction site and used in the evaluation of pharmacological properties and the link between chemical structure and biological activity^31^. Modeling and docking studies were conducted to understand the interactions of the target proteins with the compounds identified in the studied essential oil which in turn gives information about the activity of the psychrophilic enzyme in comparison with its counterparts^32^. However, no published reports exist on the phytochemistry, antioxidant properties and molecular docking of AMEO.

These lacks in knowledge leads us to explore the phytochemical, physicochemical properties, phenolic content, antioxidant activity using the FRAP (ferric reducing antioxidant power) assay and DPPH radical scavenging activity and molecular docking simulations were carried out with AMEO.

## Experimental

### Plant material

Aerial parts of *Abies marocana* were harvested from Mount Tazaout, Chefchaouen, Morocco (35°16’19.97” N, 5°06’53.14” W) at an altitude of 1721 m. The required collection permissions were obtained. We confirm that the collection of *Abies marocana* complied with all relevant institutional, national, and international guidelines and legislation. Furthermore, the study was conducted in accordance with the IUCN Policy Statement on Research Involving Species at Risk of Extinction and the Convention on International Trade in Endangered Species of Wild Fauna and Flora (CITES). After collection, the samples were dried for three days at a controlled temperature of 45 ± 2 °C. Identification and authentication were performed by Dr. Mohamed Kadiri, a botanist at the Laboratory of Applied Botany, Abdelmalek Essaâdi University, Morocco. A voucher specimen of *Abies marocana* was deposited in the Herbarium of the Biology Department, Faculty of Sciences, Abdelmalek Essaâdi University, Morocco.

### Isolation of AMEO

The air-dried plant material was subjected to steam distillation for 3 h at the Cooperative Aghsane Mechrouha. The extracted essential oil was then stored in a sealed glass vial at 4–6 °C in a refrigerator until testing and analysis. Additionally, the yield result was calculated using Eq. (1), as shown below:

1 $$\:\text{Content of essential oil (yield, \%)} = \frac{\text{volume of essential oil (mL)}}{\text{mass of dry plant (g)}}\times \mathrm{100}$$

### Analysis by GC/MS

Gas chromatography-mass spectrometry (GC-MS) was performed using a Perkin Elmer Clarus 580 series Chromatograph coupled to a Perkin Elmer SQ8S Quadrupole Mass Spectrometer. Separation was accomplished on Rtx-5MS capillary column (30 m x 0.25 mm x 0.25 μm) from Restek Corporation. Compounds were transferred into GC column via a split/splitless injector heated at 250 °C using split ratio at 20%. Pure Helium (99.99%) was chosen as a carrier gas with a constant flow rate of 1 mL.min^− 1^. The chromatographic runs were carried out under a temperature program: 40 °C for 2 min, from 40 °C to 200 °C at 6 °C/min and holds for 2 min, then heated once more to 280 °C at 6 °C/min and holds for 6 min. The mass spectrometer parameters were as follow: Ionization of the sample components was performed in Electronic Impact (EI) mode (70 eV), transfer line at 250 °C. Data acquisition was in a full scan mode (mass range m/z 50–500, and were analyzed using TubroMass software from Perkin Elmer Incorporation (Part Number 09931016, Release B, Version 6.1 2014). The compounds were identified by comparing their retention times with those of the standards under the same chromatographic conditions, and the fragmentation showed in their mass spectra with those found in the data stored in the National Institute of Standards and Technology (NIST) compounds. LabSolutions (version 2.4) was used for data collection and processing.

### Determination of physicochemical properties and organoleptic profiles

#### Determination of density

Density of AMEO was measured using a digital densimeter (Mettler Toledo, DA-100 M).

#### Determination of refractive index

Refractive index (RI) was measured using a CETI Quartz refractometer at 20 °C. The principle of operation for this refractometer based on the angular deviation produced when light is refracted.

#### Determination of optical rotation

Optical rotation (OR) was measured with a CETI Polaris polarimeter. Degrees of circumference (°) are used to express optical rotation.

#### Color and odor

Oil color was analyzed based on visual observation, and odor was evaluated by direct smelling of paper strips containing the oil.

### Measurement of total phenolic contents of AMEO

The total phenolic content of the sample was using an adapted Folin-Ciocalteu method^33^ with a few modifications. This assay is based on quantifying the total concentration of hydroxyl groups present in the sample. Briefly; 0.05 mL of the sample and made up to the volume of 0.45 mL with distilled water. then 0.25 mL of Folin-Ciocalteu reagent diluted 2 times was added and the contents mixed thoroughly. After 5 min, 1.25 mL of sodium carbonate (20%) was added. The tubes were shaken and stored in the dark for 40 min at normal temperature. The absorbance is measured at 725 nm using a Thermo Scientific TM Evolution 260 UV–Vis spectrophotometer. A calibration curve of gallic acid standards was formulated at concentrations ranging from 0.03 to 1 mg/mL under identical conditions. The total polyphenol content in extracts is expressed as milligrams of gallic acid equivalent per gram of essential oil (mg GAE/g EO). Measurements were performed in triplicate. The GAE was obtained using the following linear Eq. (2) for the standard curve:

2 $$\:y=1.1969x+0.1114\:\:\:{R}^{2}=0.9935$$

where y and x represent the absorbance and concentration (mg/mL) of GA, respectively.

### Measurement of antioxidant activity using the DPPH method

The antioxidant activity of AMEO was tested using 1,1-diphenyl-2-picrylhydrazyl (DPPH) free radical scavenging according to the spectrophotometric method of Blois^34^ as described by Brand-Williams. Briefly, 1 mL of the DPPH radical (1 mM dis- solved in methanol) is transferred into tubes containing 3 mL of different concentrations of the studied essential oil (2 to 80) mg/mL diluted in methanol. After vortex mixing, the tubes were placed in the dark at room temperature for 30 min then the absorbance was read using a spectrophotometer at 517 nm. A synthetic antioxidant, butylated hydroxytoluene (BHT), was prepared with the same procedure and used as a standard antioxidant and positive control. Each experiment was performed in triplicate. The percentage of the free radical inhibition was calculated using the following Eq. (3):

3 $$\:\mathrm{D}\mathrm{P}\mathrm{P}\mathrm{H}\:\mathrm{s}\mathrm{c}\mathrm{a}\mathrm{v}\mathrm{e}\mathrm{n}\mathrm{g}\mathrm{i}\mathrm{n}\mathrm{g}\:\mathrm{a}\mathrm{c}\mathrm{t}\mathrm{i}\mathrm{v}\mathrm{i}\mathrm{t}\mathrm{y}\:{\%}\:=\frac{(\mathrm{A}\mathrm{c}-\mathrm{A}\mathrm{s})}{\mathrm{A}\mathrm{c}}\times\:100$$

where: Ac is the absorbance of diluted DPPH, As is the absorbance of the sample with DPPH.

### Measurement of antioxidant activity using the FRAP assay

The antioxidant activity of AMEO was also tested using the ferric reducing antioxidant power (FRAP assay) as described by Oyaizu^35^. Thus, 2.5 mL of the sample at different concentrations (1.25 to 10) mg/mL diluted in methanol was mixed with 2.5 of phosphate buffer (0.2 M, pH 6.6) and 1% potassium ferricyanide (K_3_ [Fe(CN)_6_]) and then incubated at 50 °C for 20 min before adding 2.5 ml of 10% trichloroacetic acid (w/v). The mixture was centrifuged at 3000 rpm for 10 min, and 2.5 mL of the upper layer was mixed with 2.5 mL of distilled water and 100 µL of 0.1% ferric chloride (FeCl_3_). Finally, the absorbance of the mixture was measured spectrophotometrically at 700 nm, and the results were expressed as IC_50_ (mg/mL) values. For comparison, the same procedure was applied, using a methanolic solution of Rutin as a positive control. All experiments were carried out in triplicate.

### Molecular docking methodology

In this study, molecular docking calculations were performed using AutoDock Vina^36^ to model the complex formation between seven natural compunds identified in AMEO, *S*-limonene, *R*-limonene, camphene, (+)-*α*-pinene, (-)-*α*-pinene, (+)-*β*-pinene and (-)-*β*-pinene. These are found in high concentration in AMEO and thus, their contribution to the antioxidant activity of the essential oil is important to understand. Thus, carefully selected enzymes were employed to assess the antioxidant activity of the ligands. The geometry of the ligands were optimized by using the B3LYP method^37^ combined with the 6-31G(d, p)^38^ basis set using the Gaussian 16 software package^39^. Two enzymes were selected as target proteins, NAD(P)H oxidase (PDB ID: 2CDU)^40^ and nitric oxide synthase (PDB ID: 6NGI)^41^ both of them are used to asses antioxidant compounds and the corresponding crystal structures were retrieved from the RCSB Protein Data Bank (https://www.rcsb.org)^42^. For 2CDU, docking was performed using grid box parameters of 126 × 126 × 126 Å and x, y and z values of 10.141, 0.694 and 6.107 Å, respectively. For 6NGI, the grid box parameters were 126 × 126 × 126 Å and x, y and z values of 117.347, 249.585 and 359.605 Å, respectively. The exhaustiveness level was set to 8 for both docking studies. During the preparation of the receptor input file, all water molecules were erased. The corresponding ligand and protein files (PDBQT) were prepared using AutoDock Tools^43^. The results obtained from docking were analyzed using LigPlot +^44^ and PyMOL^45^. These tools provide visualization and analysis of the ligand-protein interactions, allowing for a more detailed understanding of the binding mode and potential binding sites. For each ligand, up to nine binding modes were determined. The binding modes with the lowest binding affinities (E_A_) were selected for analysis.

## Results and discussion

### Evaluation of AMEO yield

The obtained yield of the extracted AMEO was 0.24%. Since no studies were performed for the Moroccan species, this yield was compared to previous studies on other species from different countries. Our oil exhibited a lower yield than that reported by Hong et al.^46^, who found essential oil yields of 0.8 and 0.6% for *Abies holophylla* and *Abies koreana* from Korea, respectively. Additionally, a recent study^47^ reported that *Picea Abies* growing wild in Romania had a higher essential oil content, ranging from 0.95 to 1.15%, when extracted by hydrodistillation. Another study on the essential oils from tew different *Abies* concolor trees, native to western North America, reported yields of 0.737 and 0.895%^48^. Leaves of *Abies pindrow* were collected from India and the yield of the essential oil was (0.16%)^49^. The variation in essential oil yield can be attributed to several factors, including species differences, extraction methods, plant parts used, as well as geographical and climatic conditions^50,51^.

### Chemical composition of AMEO

Gas chromatography coupled with mass spectrometry (GC-MS) was employed to analyze the qualitative and quantitative composition of chemical compounds present in the essential oil.

A total of 49 compounds were identified in the essential oil, which represent 97.10% of the total composition (Table 1). Monoterpene hydrocarbons dominated in the chemical composition of AMEO. The relative percentage of the individual components was calculated based on GC peak area. The major constituents of oil were limonene (38.46%), *α*-pinene (21.16%), *β*-pinene (12.92%), and camphene (11.23%). Among these, limonene was the predominant component (Table 1).

**Table 1:** Chemical composition (relative area percentage) of AMEO by GC–MS.

No.	Compounds	MF	MW (g/mol)	RI	RI Lit^52^	RT (min)	Area (%)
1	Santene	C_9_H_14_	122	887		6.99	0.45
2	Tricyclene	C_10_H_16_	136	925		7.98	0.83
3	*α*-Pinene	C_10_H_16_	136	929	929	8.83	*21.16*
4	Camphene	C_10_H_16_	136	952	952	9.14	*11.23*
5	*β*-Pinene	C_10_H_16_	136	987	979	9.95	*12.92*
6	*β*-Myrcene	C_10_H_16_	136	991		10.05	1.85
7	Sabinene	C_10_H_16_	136	974		10.32	0.01
8	Limonene	C_10_H_16_	136	1004	1030	11.73	*38.46*
9	*γ*-Terpinene	C_10_H_16_	136	1060		11.95	0.20
10	Isoterpinolene	C_10_H_16_	136	1086		12.61	1.39
11	Linalool. formate	C_11_H_18_O_2_	182	1215		12.78	0.04
12	*Cis-*Verbenol	C_10_H_16_O	152	1142		13.16	0.03
13	*β-*Terpinene	C_10_H_16_	136	1028		13.35	0.05
14	*α-*Campholenal	C_10_H_16_O	152	1125		13.44	0.06
15	*(E)-*Pinocarveol	C_10_H_16_O	152	1139		13.78	0.07
16	Sabinol	C_10_H_16_O	152	1143		13.90	0.06
17	Linderol	C_26_H_30_O_5_	422	1166		14.45	0.07
18	Isocamphopinone	C_10_H_16_O	152	1173		14.63	0.02
19	*L*-4-terpineol	C_10_H_18_O	154	1182		14.69	0.29
20	Criptone	C_9_H_14_O	138	1184		14.95	0.22
21	Anethole	C_10_H_12_O	148	1286		15.17	0.10
22	l-Verbenone	C_10_H_14_O	150	1204		15.50	0.05
23	2-Nonyl acetat	C_11_H_22_O_2_	186	1211		15.81	0.07
24	Carvone	C_10_H_14_O	150	1242		16.26	0.02
25	Linalyl acetate	C_12_H_20_O_2_	196	1257		16.45	0.22
26	2,5-Diethylphenol	C_10_H_14_O	150	1271		17.71	0.04
27	Methyl geranate	C_11_H_18_O_2_	182	1323		18.04	0.04
28	*α-*Terpinyl acetate	C_12_H_20_O_2_	196	1350		18.75	1.16
29	Isogeraniol	C_10_H_18_O	154	1240		19.11	0.03
30	*α-*Copaene	C_15_H_24_	204	1396		19.31	0.04
31	*γ-*Muurolene	C_15_H_24_	204	1477		19.60	0.05
32	Methyleugenol	C_11_H_14_O_2_	178	1402		19.93	0.19
33	*α-*Himachalene	C_15_H_24_	204	1449		20.01	0.10
34	Caryophyllene	C_15_H_24_	204	1419		20.39	1.36
35	*β*-copaene	C_15_H_24_	204	1481		20.48	0.06
36	Humulene	C_15_H_24_	204	1454		21.08	0.67
37	Dodecyl alcohol	C_12_H_26_O	186	1473		21.31	0.09
38	Curcumene	C_15_H_22_	202	1483		21.54	0.01
39	*D-*Amorphene	C_15_H_24_	204	1389		21.85	1.99
40	*α-*Himachalene	C_15_H_24_	204	1449		22.07	0.04
41	*γ-*Cadinene	C_15_H_24_	204	1513		22.29	0.33
42	Cubenene	C_15_H_24_	204	1532		22.65	0.04
43	*α-*Calacorene	C_15_H_20_	200	1542		22.86	0.01
44	*trans-*Chrysanthemal	C_10_H_16_O	152	1153		23.37	0.02
45	Caryophylene oxide	C_15_H_20_O	220	1581		23.74	0.34
46	Cubenol	C_15_H_26_O	222	1642		24.28	0.03
47	Cedrelanol	C_15_H_26_O	222	1640		24.84	0.48
48	ent-Pimara-8,15-diene	C_20_H_32_	272	1902		29.67	0.04
49	Abietadiene	C_20_H_32_	272	2080		33.12	0.07
Number of compounds identified							49
Total identified (%)							97.10
Monoterpene hydrocarbons (%)							88.55
Oxygenated monoterpenes (%)							2.59
Sesquiterpène hydrocarbons (%)							4.81
Oxygenated sesquiterpenes (%)							1.15

MF molecular formula, MW molecular weight, RI retention index, RT retention time.

As mentioned above, no studies on AMEOs have been found in the literature, its composition was compared with that of other *Abies* species. Previous research indicates that the chemical composition of *Abies* essential oils varies significantly between species. For example, Ramdani et al.^51^ reported that the essential oil extracted from aerial parts of *Abies numidica* (an endemic species of Algeria) was mainly composed of *α*-pinene (22.6%), limonene (19.7%), *β*-pinene (12.3%), camphene (11.2%) and *β*-phellandrene (7.8%). Similarly, *Abies Alba* essential oil from Korea contained 20 compounds, with bornyl acetate (30.31%) as the dominant component, followed by camphene (19.81%), 3-carene (13.85%), tricyclene (12.90%), di-limonene (7.50%), *α*-pinene (2.87%)^53^. Additionally, the essential oil compositions of two different mature trees of *Abies concolor* collected from Kuna, which showed *α*-pinene (20.5–15.2%), camphene (7.5–10.2%), *β*-Pinene (25.6–24.2%), δ-3-carene (6.5–5.5%), limonene (6.9–5.4%), and bornyl acetate (14.6–22.1%)^48^. In another study, the essential oil extracted from the needles and twigs of *Abies koreana* from Korea contained 36 compounds, representing 98.67% of the total composition. The major compounds identified were bornyl ester (41.79%), camphene (15.31%), *α*-pinene (11.19%), limonene (8.58%), fenchyl acetate (5.55%), and terpinene (2.29%)^46^. Compared to these species, AMEO exhibited a distinct chemical profile, with limonene as the predominant compound. In the contrast, the essential oil of *Abies numidica* was mainly dominated by *α*-pinene and limonene; *Abies alba* essential oil characterized by bornyl acetate and camphene; *Abies concolor* showed high levels of *β*-pinene, bornyl acetate and *α*-pinene; while *Abies koreana* essential oil was primarily composed of bornyl acetate, camphene and *α*-pinene. These variations in chemical composition can be attributed to differences in plant material, extraction methods, distillation duration and environmental factors such as geographical location and climatic conditions. Numerous studies have shown that these factors can significantly influence the chemical profile of essential oils^14^.

### Physicochemical analysis

Organoleptic and physicochemical analysis are valuable methods in determining the quality of essential oils^26,54^. These evaluations are crucial for determining whether the quality of oil tested (density, refractive index and optical rotation). Density measurement was determined to be (0.896 ± 0.010) g/cm^3^, refractive index was found to be (1.470 ± 0.010) and optical rotation was (-40.52 ± 0.04)°. Furthermore, the values of these properties, density, refractive index, optical rotation, color and odor of the essential oil were good agreement according to ISO 10869:2010, the essential oil of Siberian fir (*Abies sibirica Ledeb*), originating from Russia, is characterized by specific chemical markers that determine its purity and effectiveness, as shown in Table 2^55^.

**Table 2:** Physico-chemical characteristics of AMEO in comparison to ISO 10869:2010 requirements.

Physico-chemical characteristics	Value	ISO 10869:2010
Density (g/cm^3^)	0.896 ± 0.010	–
Refractive index	1.470 ± 0.010	1.468 to 1.473
Optical rotation (°)	− 40.52 ± 0.04	− 25 to -40
Color	Colorless	Colorless to pale yellow
Odour	Freshly	Freshly cut wood, resinous.

Values in the table are means of three independent experiments and error bars indicates standard deviation of the mean.

‘–’ indicates value that was not determined.

### Evaluation of total phenolic contents (TPC)

Phenolic compounds represent a diverse class of antioxidants characterized by the presence of benzene rings and hydroxyl groups in their structures which act as free radical terminators^56^. In this study, a spectrophotometric methodology was used to evaluate the TPC of AMEO. To the best of our knowledge, no previous studies have documented the TPC of the AMEO. The quantified TPC value in the tested oil was (5.07 ± 0.49) mg GAE/g EO.

Comparing TPC of various extracts provides a valuable foundation for understanding the phenolic composition across different plant species. In this study, the TPC of the tested oil was found to be higher than that reported for etheric extracts from various parts of *Abies marocana*, including twigs, needles, and cones^13^, yet lower than the TPC values recorded in ethanolic extracts of fresh *Picea Abies* buds from Romania^3^. When compared to essential oils from other plants, the TPC of *Pinus* taxa needles collected from wild populations in China ranged between 26.50 and 60.01 mg GAE/g EO^57^. Notably, the TPC of our essential oil was significantly higher than that of *Pistacia lentiscus* essential oils from Tunisia, which ranged from (2.99 ± 0.28) to (0.48 ± 0.14) mg GAE/g EO, depending on the collection site^58^. In addition, AMEO was notably lower TPC than recent findings on *Pistacia lentiscus* essential oils^52^. Additionally, the TPC of AMEO was lower than that of *Syzygium cumini* essential oil, which reached (12.8 ± 0.10) mg GAE/g EO^59^. Generally, medicinal plants are rich sources of natural antioxidants, with phenolic compound concentrations influenced by genetic and environmental factors^4^. These variations may be attributed to the genotype of each subspecies tested, as well as to environmental factors^56^.

### Evaluation of antioxidant activity

Antioxidant activity is one of the most valuable biological properties, playing a crucial role in industries such as cosmetics, food, and beverages. It is particularly significant due to its essential function in maintaining the balance of biological systems. The antioxidant potential of AMEO was evaluated using two methods: the DPPH radical scavenging and the FRAP (ferric reducing antioxidant power) assay.

Table 3 present the result of the DPPH radical scavenging effect of the sample, which was determined to be (136.28 ± 7.8) mg/mL. However, this value indicates a weak radical scavenging capacity compared with the synthetic antioxidant BHT (0.11 ± 0.004) mg/mL. Thus, AMEO exhibits limited efficacy against DPPH free radicals.

**Table 3:** Antioxidant activity by (DPPH and FRAP assays) of AMEO.

	Activity on DPPH IC_50_ (mg/mL)	FRAP IC_50_ (mg/mL)
Essential oil	136.28 ± 7.80 ^a)^	1.47 ± 0.04 ^a)^
BHT	0.110 ± 0.004 ^b)^	–
Rutin	–	0.040 ± 0.001 ^c)^

Values in the table are means of three independent experiments and error bars indicates standard deviation of the mean.

^a^Refers to the essential oil sample, ^b^Standard compound for DPPH assay, ^c^Standard compound for FRAP assay.

Few studies have focused on evaluation of the antioxidant properties of essential oils derived from the *Abies* species. For example, the essential oil of *Abies pindrow*, naturally growing wild in Kashmir, demonstrated a significant free radical scavenging activity against DPPH (0.008 mg/mL)^49^. In contrast, the IC_50_ value reported for *Abies numidica* essential oil (26.70 ± 9.02) mg/mL ^60^ was significantly higher than that observed in our study. Moreover, the essential oil of silver fir (*Abies alba*) shows strong antiradical activities against DPPH, with an IC_50_ value of 0.027 mg/mL ^53^. Findings from *Abies numidica* needles shows a weak antioxidant potential, while essential oil extracted from its leaves demonstrated a significantly stronger DPPH free radical scavenging capacity (0.288 ± 0.024) mg/mL ^51^, This may be attributed to the presence of *β*-caryophyllene as the major compound, which contributes to the higher antioxidant activity, as demonstrated in the study by Sobrinho et al.. ^51^ reported that *β-*caryophyllene exhibited mild antioxidant potential in the DPPH scavenging assay. On the other hand, The IC_50_ value of essential oil of *Abies balsamea* needles was so low that its IC_50_ value could not be determined^61^. Our essential oil was characterized by *α*-pinene, *β*-pinene, camphene, and limonene as the main compounds. Moreover, some studies have demonstrated that monoterpenes (limonene, *α*-pinene, camphene and *β*-pinene) exhibit low antioxidant effectiveness when tested individually^62,63^.

The reducing power activity of our sample, specifically their ability to reduce ferric ions (Fe³⁺) to ferrous ions (Fe^2+^), is presented in Table 3. IC_50_ Value of the positive control Rutin was determined to be (0.04 ± 0.001) mg/mL, indicating significantly higher antioxidant activity compared to the tested oil (1.47 ± 0.04) mg/mL. When comparing our findings with previous studies on essential oils from different plants, *Abies numidica* essential oil from Algeria exhibited a stronger antioxidant activity, with an IC_50_ value of (0.16 ± 0.02) mg/mL^60^. Moreover, the tested essential oil of *Eucalyptus globulus* from Algeria exhibited a very weak antioxidant capacity, with an IC_50_ value of (115.39 ± 1.45) mg/mL^64^, demonstrates a very weak correlation with the findings of our sample. Additionally, the three *Pistacia lentiscus* essential oils showed a stronger ability to reduce Fe³⁺ to Fe^2+^ compared to AMEO, Fnideq *Pistacia lentiscus* essential oil exhibited the highest antioxidant capacity, with an IC₅₀ value of (0.04 ± 0.01) mg/mL, followed by Masmouda at (0.29 ± 0.02) mg/mL, while Zinat demonstrated the lowest potency at (0.57 ± 0.02) mg/mL^52^, this may be attributed to the high levels of phenolic compounds in their essential oils, as previously observed. According to Hazzit in 2009^60^, essential oils rich in thymol and hydroxyl groups exhibit greater reductive power than those with lower phenol content. The presence of phenolic compounds in our essential oil, quantified at (5.07 ± 0.49) mg GAE/g EO, may explain the observed reducing power.

The divergence between DPPH and FRAP results reflects the distinct mechanisms of these assays. DPPH measures the ability of compounds to neutralize free radicals directly, whereas FRAP evaluates electron-donating ability^65^. Thus, AMEO shows weak radical scavenging but moderate electron-donating ability, consistent with its chemical composition: monoterpenes contribute to reducing power along with the present phenolics which also enhance FRAP response.

### Molecular Docking

Molecular docking proves to be a valuable tool for better understanding the interaction of bioactive agents with biomolecules^66^, for assessing the antioxidant potential of compounds through the analysis of their binding interactions with target proteins^67^.

In this study, molecular docking was performed to evaluate the binding affinity of seven natural ligands from AMEO with two selected antioxidant targets. The docking results were assessed based on the lowest binding affinities obtained for each ligand. The results in Tables 4 and 5 show the tendency of each extracted structure to bind to the active site of the studied proteins.

**Table 4:** Docking score of ligands with antioxidant virulence associated with NAD(P)H oxidase (PDB ID: 2CDU).

Ligand	Binding affinities (E_A_, kcal/mol)	Residues involved in hydrophobic interactions
*R*-limonene	− 6.0	HIS10, THR9, ASP282, ALA280, ALA11, THR112, GLY114, GLY281 and ALA303
*S*-limonene	− 6.2	HIS10, THR9, ALA11, GLY12, THR112, GLY114, ASP282, ALA300 and ALA303
camphene	− 5.6	THR13, LYS17, PHE14, VAL304, HIS10 and TYR62
(+)-*α*-pinene	− 5.2	ASP282, THR9, SER41, LYS134, ALA300 and ALA303
(-)-*α*-pinene	− 5.2	LYS134, HIS10, SER41, THR9, ALA11, ASP282, GLY114 and SER115
(+)-*β*-pinene	− 4.7	VAL368, PRO290, ASN289, ILE409, LEU388, LYS412 and THR384
(−)-*β*-pinene	− 5.4	GLY114, THR112, THR9, ALA303, ALA300, ASP282, ALA11, HIS10 and GLY12

**Table 5:** Docking score of ligands with antioxidant virulence associated with nitric oxide synthase (PDB ID: 6NGI).

Ligand	Binding affinities (E_A_, kcal/mol)	Residues involved in hydrophobic interactions
*R*-limonene	− 7.1	PHE589, LEU429, SER590, GLY590, PHE709 and TRP414
*S*-limonene	− 7.2	PHE589, SER590, GLY590, PHE709, LEU429 and TRP414
camphene	− 5.1	ARG419, ALA417, VAL572, PHE709, MET575, CYS420 and PHE589
(+)-*α*-pinene	− 5.8	PHE589, TRP592, CYS420, SER590, TRP414 and GLY591
(−)-*α*-pinene	− 5.5	GLY591, SYC420, TRP414, PHE589 and SER590
(+)-*β*-pinene	− 5.3	TYR711, MET575, ARG704, PHE709, ALA417, SER418, ASN416, ARG419, SER708 and GLU710
(−)-*β*-pinene	− 5.3	GLY591, TRP414, CYS420, PHE589 and TRP592

Due to the variations in the number of selected docking poses for each ligand-protein complex the optimal binding structure differed among ligands. Both, *R*-limonene and *S*-limonene showed the strongest binding for the targets (= > 6 kcal/mol), with stronger binding affinities for nitric oxide synthase compared to NAD(P)H oxidase (− 7.1 and − 7.2 kcal/mol, respectively).

**Fig. 1:**
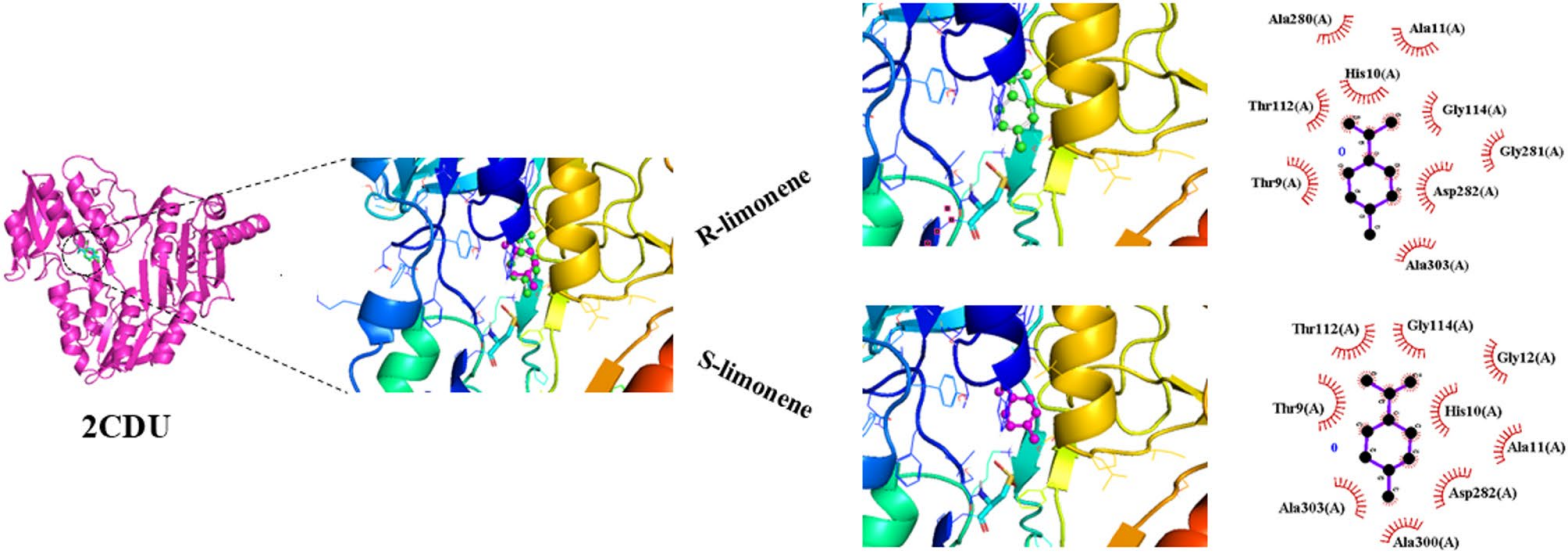
Ligands docked in the active site of NAD(P)H oxidase (PDB ID: 2CDU). Interactions are also shown for *R*-limonene and *S*-limonene as ligands. Non-bonded contacts were defined using a 3.90 Å maximum distance.

Studies on the structure of NAD(P)H oxidase have shown that residues in the active site including HIS10, THR9, ASP282, ALA280, ALA11, THR112, GLY114, GLY281 and ALA303 contribute significantly to the binding affinity and stability of the complex formed with *R*-limonene. In the case of *S*-limonene, the same residues were also involved except GLY281, but two new interacting units, GLY12, and ALA300 were also identified (Fig. 1). These residues collectively facilitate effective binding through hydrophobic contacts between the ligand and surrounding nonpolar residues.

**Fig. 2:**
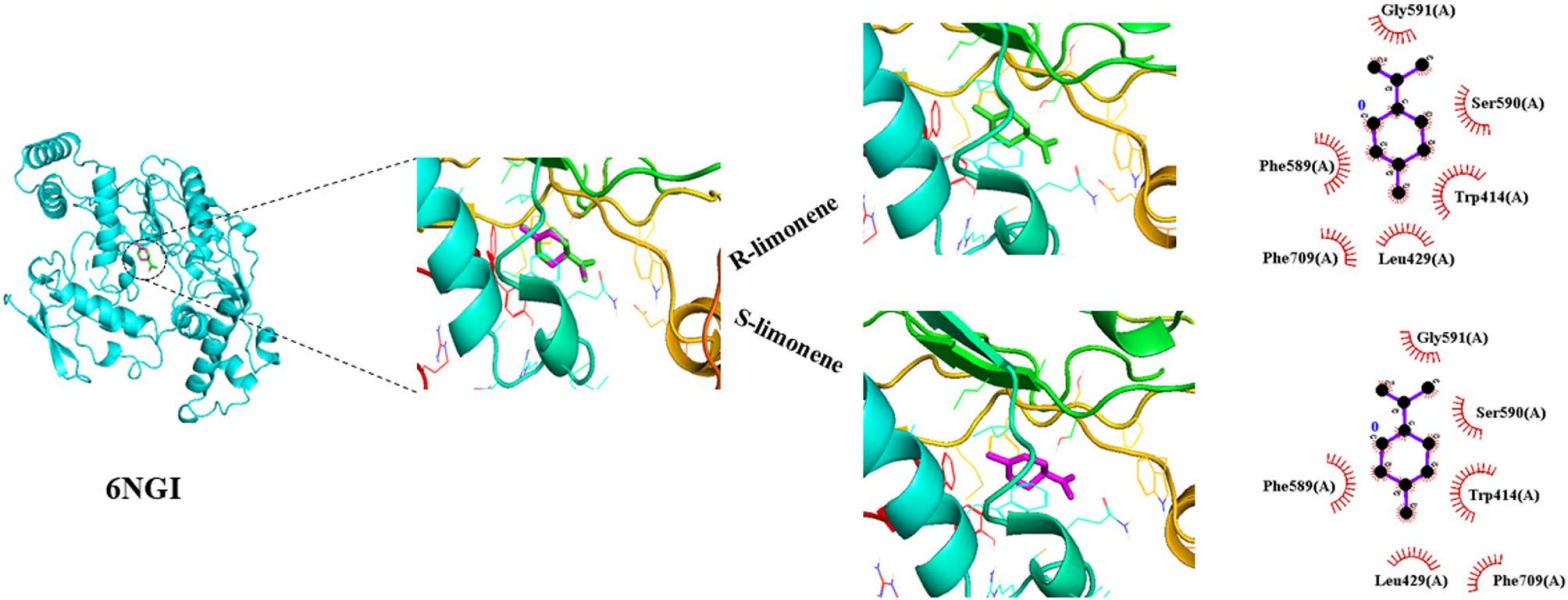
Ligands docked in the active site of nitric oxide synthase (PDB ID: 6NGI). Interactions are also shown for *R*-limonene and *S*-limonene as ligands. Non-bonded contacts were defined using a 3.90 Å maximum distance.

In case of the nitric oxide synthase target, PHE589, LEU429, SER590, GLY590, PHE709 and TRP414 were involved in the interaction with *R*-limonene, and also played a significant role in binding *S*-limonene (Fig. 2). In all interactions analyzed for both proteins, binding was established by hydophobic contacts. All in all, among the seven studied compounds, *R*-limonene and *S*-limonene emerged as the most promising antioxidants due to their favorable binding profiles with both target proteins.

## Conclusions

The phytochemical composition, physicochemical properties, total phenolic content and antioxidant activity of the essential oil of *Abies marocana* from Mont Tazaout have been successfully explored for the first time. The investigation of AMEO highlights its phytochemical richness, dominated by limonene, *α*-pinene, *β*-pinene, and camphene. Despite weak radical scavenging in the DPPH assay (136.28 ± 7.8 mg/mL), AMEO demonstrated notable reducing power in the FRAP test (1.47 ± 0.04 mg/mL) and measurable phenolic content. Docking studies further revealed *R*- and *S*-limonene as promising compounds with favorable interactions with NAD(P)H oxidase and nitric oxide synthase. These results suggest a limited antioxidant capacity, which may be due to the dominance of monoterpene hydrocarbons and the limited presence of phenolic compounds. However, further research is needed to explore its full biological properties and potential synergistic effects, such as the antimicrobial and antioxidant potential of individual compounds can be found in essential oils derived from different parts of *Abies marocana*.

## Data Availability

Data is provided within the manuscript or supplementary information files.
